# Defect rate prediction and failure‐cause diagnosis in a mass‐production process for precision electric components

**DOI:** 10.1002/ansa.202300019

**Published:** 2023-05-07

**Authors:** Hiromasa Kaneko

**Affiliations:** ^1^ Department of Applied Chemistry School of Science and Technology Meiji University Kawasaki Japan

**Keywords:** defect rate, ensemble learning, fault diagnosis, feature importance, random forests

## Abstract

Many defects occur during the mass production of precision electrical components. To control and manage them, process variables (PVs), such as the temperature, pressure, flow rate, and liquid level, are measured and time‐series data analyzed. However, identification of point of defects is difficult as any operation can cause defects and multiple equipment units are used in parallel for some operations. This study considers the combination of unfavourable conditions between operations to predict the defect rate (DR) of products. A dataset measured in an actual mass‐production process for precision electrical components is analysed to predict the DR of the products. Data analysis is performed on a dataset generated from an actual mass‐production process for precision electrical components, and machine learning models. are constructed using ensemble learning methods, such as random forests, the gradient boosting decision tree, XGBoost, and LightGBM. Conventional univariate analyses only show a maximum correlation coefficient of 0.17 with a DR and process variables (PVs). In this study, we improved the correlation coefficient to 0.73 using a multivariate analysis, including the data of PVs that are not considered important in the process, and appropriately transformed PVs based on the domain knowledge of the process. Furthermore, PVs that were closely related to the DR could be diagnosed based on the feature importance of the constructed machine‐learning models. This study confirms the importance of using domain knowledge to improve the prediction ability of machine learning models and the interpretation of constructed models.

**List of Abbreviations**
DR: defect rate
DT: decision tree
GB: gradient boosting
GBDT: gradient boosting decision tree
ICA: independent component analysis
LGB: LightGBM
LIME: local interpretable model‐agnostic explanations
PCA: principal component analysis
PVs: process variables
RF: random forests
SHAP: Shapley additive explanations
XGB: XGBoost

## 1 INTRODUCTION

In chemical and industrial plants, process variables (PVs), such as the temperature, pressure, flow rate and liquid level, are measured, and time‐series data are used for plant control and management. For example, data on normal process conditions in a plant are used to define and model the data domain of PVs representing normal process conditions. This model is used to detect process failures in plants.^1^ Examples of linear methods are principal and independent component analyses (PCAs and ICAs, respectively).^2^, ^3^ In addition, dynamic PCAs^4^ and ICAs^5^ were modelled by adding time‐delayed variables to consider process dynamics. Nonlinear methods include kernel PCAs,^6^ k‐nearest neighbour algorithms,^7^ one‐class support vector machines,^8^ self‐organising maps^9^ and generative topographic mappings^10^ to consider the nonlinearity between PVs.

Soft sensors can be utilised when abnormal and normal data exist in plants and when difficult‐to‐measure PVs, such as the concentration and density, need to be quickly controlled. A soft sensor is a regression model constructed between easy‐to‐measure PVs, that is, x‐values, such as the temperature, pressure, flow rate and liquid level, and difficult‐to‐measure PVs, that is, y‐values, such as abnormalities, concentration and other properties. By inputting the measured x‐values in real‐time into the model, y‐values can be predicted. Based on the predicted values, anomalies can be detected and processes can be controlled. Regression analysis methods include linear methods, such as partial least squares regression^11^ and least absolute shrinkage and selection operator,^12^ and nonlinear methods include support vector regression,^13^ Gaussian process regression,^14^ deep neural networks,^15^ random forests (RF),^16^ the gradient boosting decision tree (GBDT),^17^ XGBoost (XGB)^18^ and LightGBM (LGB).^19^ Adaptive soft sensors^20^ have been developed to adapt to the changing plant process conditions.

To investigate x in relation to y, for example, to search for PVs responsible for abnormal data, the constructed regression model has to be interpreted to reveal the relationship between y and x. In local interpretable model‐agnostic explanations (LIME)^21^ and Shapley additive explanations (SHAP),^22^ which can be combined with any regression method, the slope of x with respect to y around a sample point is established by determining an approximate expression for the shape of the model at that sample point. LIME and SHAP can be used to discuss the local contribution or direction of x to y. For example, for a sample with the largest y‐value, the directionality of x can be discussed to further improve the y‐value. However, this study focuses on the feature importance, which is the degree of influence of each x on y in the entire dataset. Several methods exist for setting the feature importance in RF models,^23^, ^24^, ^25^, ^26^, ^27^, ^28^, ^29^, ^30^, ^31^ including the mean‐reduced impurity and permuted feature importance.^32^ The feature importance of the x‐value is calculated by considering the y‐value. However, it may differ among high, medium and low y‐values. Shimizu and Kaneko proposed a hybrid model of a decision tree (DT) and RF, in which the importance of the RF is calculated for each leaf node of the DT model, with DT providing a global interpretation of the entire dataset and RF providing a local interpretation for each cluster.^33^

In this study, the number of defects that occur during the mass production of precision electrical components by a Japanese manufacturer should be decreased. The process consists of 11 chemical and physical unit operations. Each operation is a stacking process, which implies that the performance of the previous operation affects the results of subsequent operations. The defects targeted in this study can be judged as significant or insignificant only after the final operation is completed, which is difficult to identify because any operation can cause defects. As the processing capacity of the equipment for each operation differs, multiple equipment units are used in parallel for some operations. In addition, different grades of products from the targeted grade are loaded onto the same equipment.

In the present process, even when each operation is performed within specifications, the final product can be defective depending on the combination of operations. Because 11 operations are involved, considering all combinations of operations is difficult. Therefore, this study considers the combination of unfavourable conditions between operations, which is not estimated by considering the mechanism in the process, by predicting the defect rate (DR) of the precision electric parts from PVs in a mass‐production process based on a chosen dataset, such as the manufacturing conditions, manufacturing date and time, operators, equipment, environmental factors, evaluations in each operation and diagnosing the causes of the high DR. The objective of this study was to contribute to process improvement with machine‐learning‐based models using routinely measured process data, including the data of PVs that were not considered important in the process, and to improve the predictive ability of machine‐learning models by utilising domain knowledge of the process. Data on PVs considered to be related and unrelated to the DR was collected, and regression analyses were performed using various methods, including RF, GBDT, XGB and LGB, which can be used to calculate the feature importance of the PVs. In addition, the predictive ability of the constructed models was verified, and the PVs related to the DR were analysed using feature importance. Finally, the PVs considered to be related to the DR were compared to those considered to be unrelated to the DR to diagnose the cause of the high DR.

## 2 MATERIALS AND METHODS

### 2.1 Data

In the mass‐production process of precision electrical components, standard approaches have been studied, such as product analyses, small‐scale experiments based on temporary system diagrams, and investigations on the causes of defects through line experiments. However, these approaches have not been able to identify defective products. Samples were collected daily for 1550 days and used as the dataset. A total of 999 samples were used, and those containing missing values were removed. The objective variable y is the DR and the explanatory variable x are the six PVs, namely A, B, C, D, E and F.

A shows the inspection data of the process‐workmanship evaluation at a relatively early stage of the process. The process is a complex chemical reaction, and the results are highly variable, which can have an impact on defects in the small‐scale verification of principles. B represents the impurity data for the auxiliary material used in the chemical reaction. Impurities in this material are estimated to remain in the product at the intermediate stage and affect subsequent processes. C shows the inspection data for the evaluation of the physical processing. This process is an intermediate step in the overall process, and the results are the accumulated results of variations in the conditions of the material and each process up to this point, which are assumed to affect the remaining processes. D shows the storage environment of the work‐in‐process in the intermediate state after a certain process. In the basic experiment, the effect on the material properties was confirmed, making it a candidate cause of defects. E and F show the data on the date and time of manufacturing, respectively, for different processes. No clear correlation was observed during the preliminary investigation.

**FIGURE 1:**
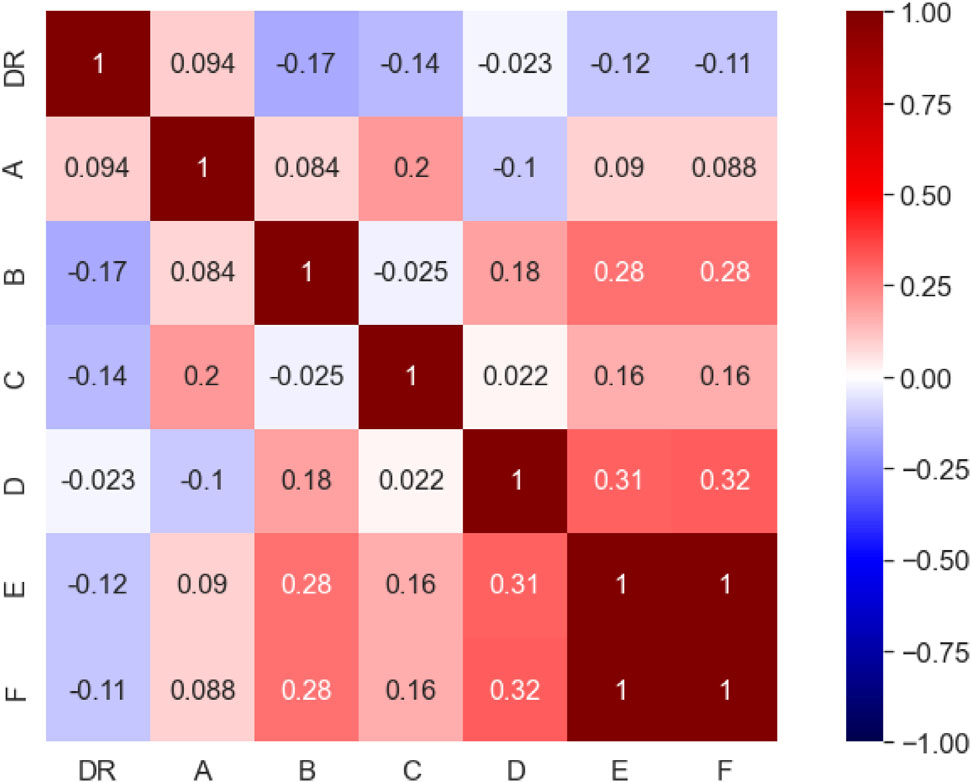
Correlation matrix between the defect rates (DRs) of A, B, C, D, E and F.

**FIGURE 2:**
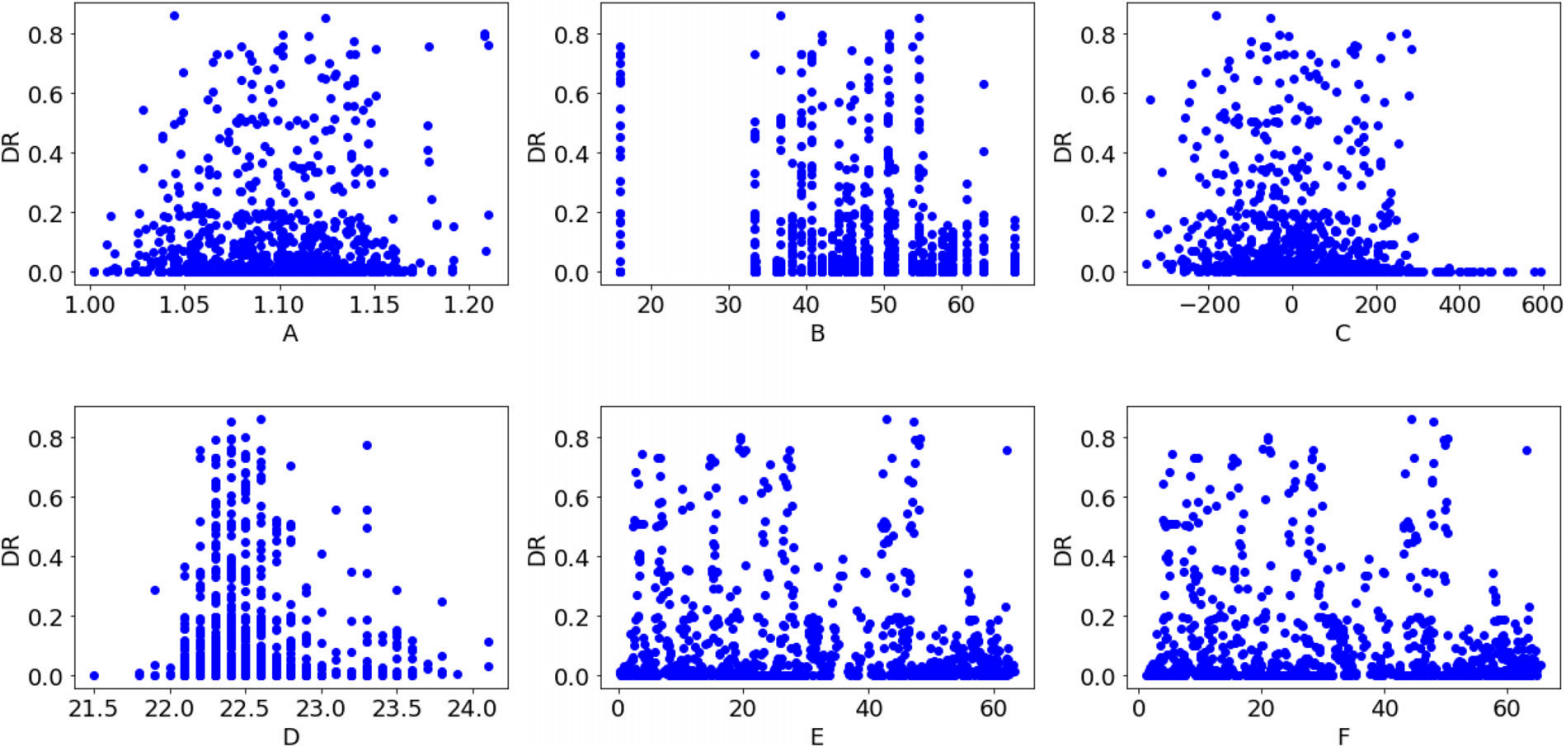
Scatter plots between the defect rates (DRs) of A, B, C, D, E and F.

The correlation matrices and scatter plots between the DRs of A, B, C, D, E and F are shown in Figures 1 and 2, respectively. The maximum absolute value of the correlation coefficients between the DRs of A, B, C, D, E and F was 0.17 for B. Although the scatter plots showed that the DR tended to be smaller when the values of C and D were large, there was no clear correlation between the DRs and x‐values.

### 2.2 Nonlinear regression method

In this study, RF, GBDT, XGB and LGB, which are used to calculate the feature importance of x, were used to determine the importance of each PV system for the DR.

RF is an ensemble learning method based on DTs. Samples and X variables were randomly selected from all datasets to construct subdatasets, and DTs were constructed for each subdataset. Because each DT was constructed using different samples and X variables, the prediction accuracy of each DT was different.

In RF,^16^ the importance of X can be calculated with respect to the *j*th sample obtained using the following equation:

(1) $$I_{j}=\frac{1}{k}\sum_{T}\sum_{t\in T,j}\frac{n_{t}}{n}\Delta E_{t}$$

where *k* is the number of subdatasets, *T* is the DT in which the *j*th sample of x is used, *t* is the node, *n* is the number of samples at node *t*, and Δ*E_t_* is the difference value of the evaluation function at node *t*. The square sum of the residuals was used as the evaluation function. The larger the *I_j_* value, the more important is the x‐value. RandomForestRegressor,^34^ from the scikit‐learn library, was used to build the RF model.

Gradient boosting (GB) is a form of ensemble learning and boosting that integrates many submodels to improve predictive ability. Submodels were constructed to improve samples with large errors in a previously constructed sum model and to reduce the following loss function:

(2) $$L\left(y^{\left(i\right)},f\left(x^{\left(i\right)}\right)\right)=\frac{1}{2}{\left(y^{\left(i\right)}-f\left(x^{\left(i\right)}\right)\right)}^{2}$$

where *y* ^(^ *^i^* ^)^ and *x* ^(^ *^i^* ^)^ are the actual y‐ and x‐values in the *i* ^th^ sample, respectively, and *f*(x^(^ *^i^* ^)^) is the y‐value predicted by submodel *f*. The gradient of the loss function is expressed as

(3) $$-\frac{\partial L\left(y^{\left(i\right)},f\left(x^{\left(i\right)}\right)\right)}{\partial f\left(x^{\left(i\right)}\right)}=y^{\left(i\right)}-f\left(x^{\left(i\right)}\right)$$

The DT is generally used in the GBs. In this study, the GradientBoostingRegressor,^35^ XGB^36^ and LGB^37^ were performed.

## 3 RESULTS AND DISCUSSION

**FIGURE 3:**
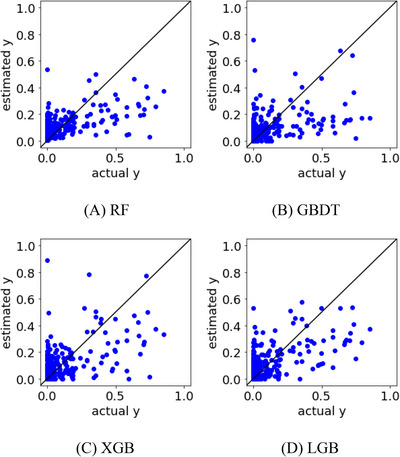
Actual versus estimated y‐values in the test data in Case Study 1 for (A) random forests (RF), (B) gradient boosting decision tree (GBDT), (C) XGBoost (XGB), and (D) LightGBM (LGB).

The 999 samples were divided into 75% training and 25% test data. Regression models were constructed using the training data to predict the test data. In Case Study 1, we analysed A, B, C and D, which were related to the DR. The correlation coefficients between the actual and estimated y‐values in the test data are shown in Table 1, and their plots are shown in Figure 3. The correlation coefficient was 0.57 for LGB, which was the best prediction result. Compared with the maximum value of 0.17 in the univariate analysis, the multivariate analysis confirmed that the predictive ability of the DR was improved. However, from the plots in Figure 3, there are scattered samples with large actual DR values even though the DR was estimated to be small, and it is desirable to reduce these samples.

**TABLE 1:** Correlation coefficients between the actual and estimated y‐values in the test data.

	**Case study 1**	**Case study 2**	**Case study 3**
RF	0.56	**0.67**	0.66
GBDT	0.40	0.56	0.53
XGB	0.51	0.65	0.59
LGB	**0.57**	**0.67**	**0.73**

Abbreviations: GDBT, gradient boosting decision tree; LGB, LightGBM; RF, random forests; XGB, XGBoost.

**FIGURE 4:**
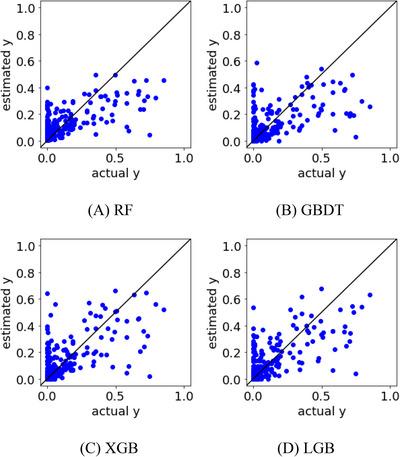
Actual versus estimated y‐values in the test data of Case Study 2 for (A) random forests (RF), (B) gradient boosting decision tree (GBDT), (C) XGBoost (XGB), and (D) LightGBM (LGB).

In Case Study 2, a regression analysis was conducted by adding E and F, which were considered unrelated to the DR, to the x‐values. The correlation coefficients between the actual and predicted y‐values in the test data are shown in Table 1, and their plots are shown in Figure 4. The correlation coefficient was 0.67 for LGB, which was the best prediction result, confirming that the addition of PVs that are unrelated to the DR improves the predictive ability of the DR prediction model. Figure 4 also shows that the samples were distributed closer to the diagonal line than those shown in Figure 3, indicating that the DR values could be predicted with high accuracy using multivariate analysis. It was confirmed that the predictive ability of the model was improved by machine learning using not only the PVs considered to be related to the y‐values but also the other PVs related to the x‐values.

**FIGURE 5:**
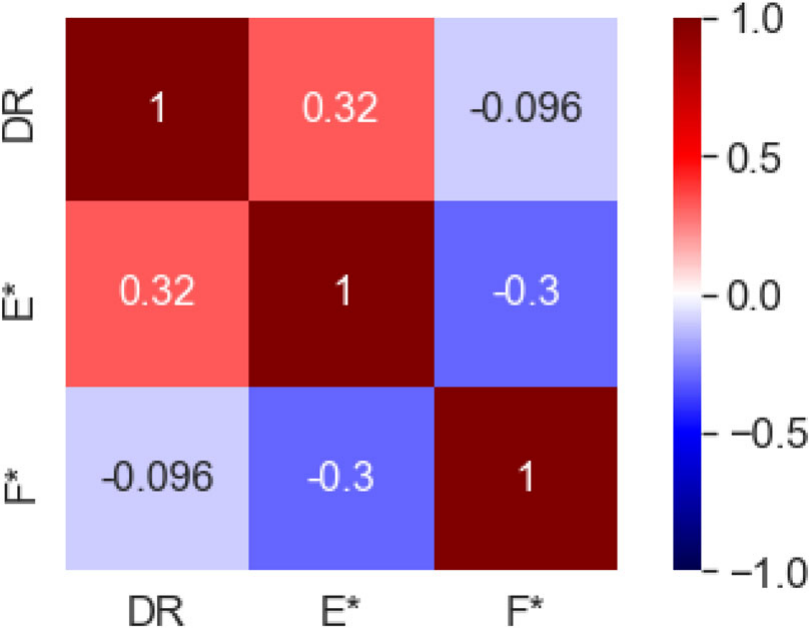
Correlation matrix between the defect rates (DRs) of E*, and F*.

**FIGURE 6:**
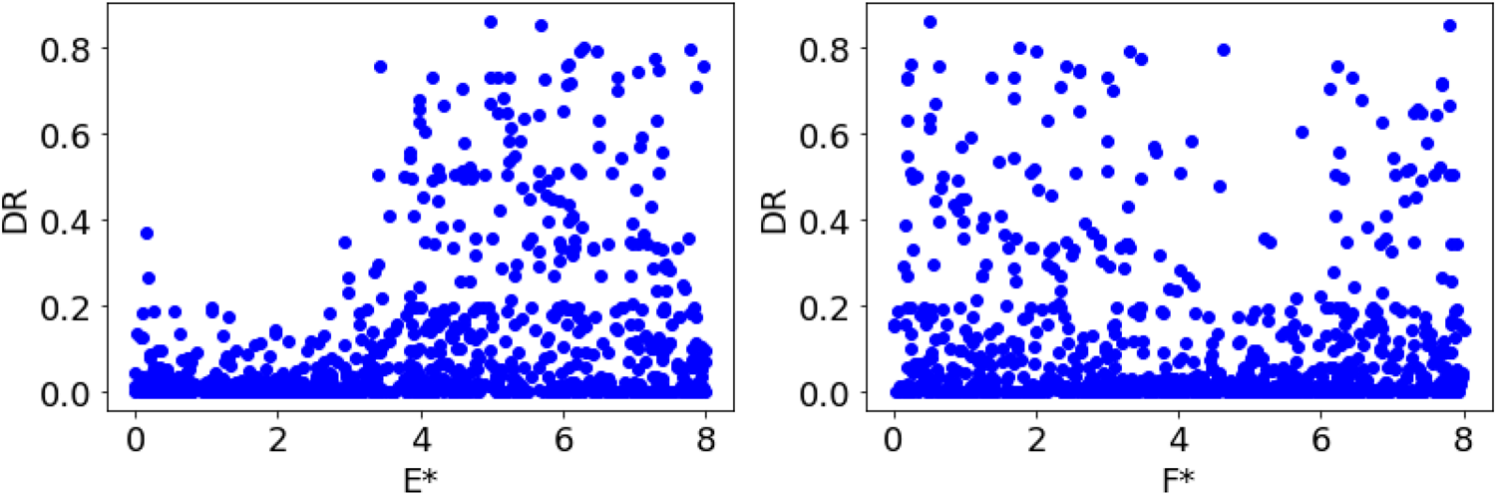
Scatter plots between the defect rates (DRs) of E* and F*.

As a result of the discussion of E and F, which were unrelated to the DR, the difference between the high and low DR values was suspected to be due to the influence of the auxiliary materials used in the process. Because the auxiliary materials were periodically replaced, E and F were converted to E* and F*, respectively, starting from the timing of the replacement of the auxiliary materials. The correlation matrices between the DRs of E* and F* are shown in Figure 5, and their scatter plots are shown in Figure 6. The absolute correlation coefficient between the DRs of E* was 0.32, which is larger than the previously reported maximum value (0.17 for B), suggesting that E* is strongly related to the DR. The scatter plot in Figure 6 also confirms the tendency of the DR to increase when E* is large. It was confirmed that the ability to explain the y‐values can be improved by appropriately transforming the PVs.

**FIGURE 7:**
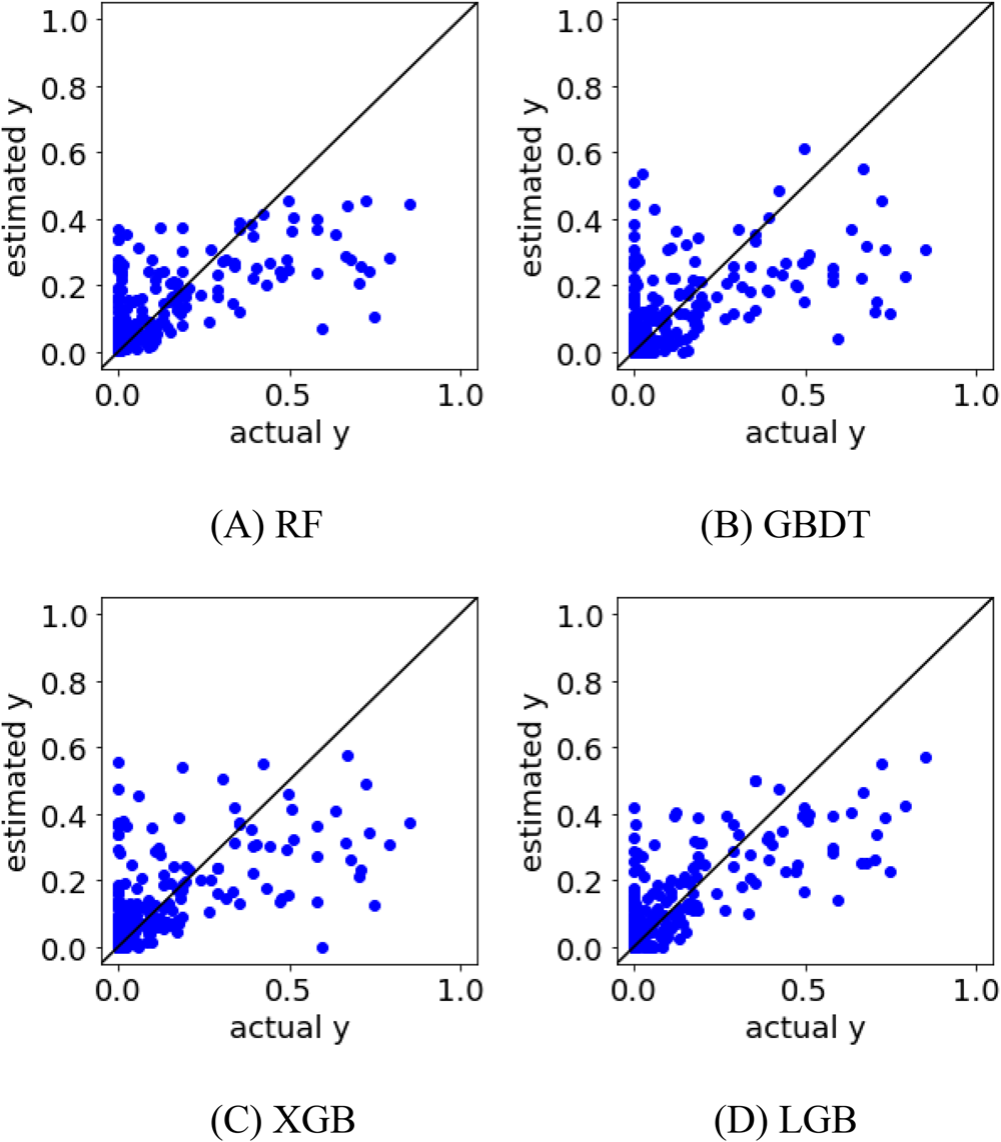
Actual versus estimated y‐values in the test data in Case Study 3 for (A) random forests (RF), (B) gradient boosting decision tree (GBDT), (C) XGBoost (XGB), and (D) LightGBM (LGB).

In Case Study 3, a regression analysis was performed with PVs, including E* and F*. The correlation coefficients between the actual and estimated y‐values in the test data are listed in Table 1, and their plots are shown in Figure 7. The correlation coefficient was 0.73 for LGB, which was the best prediction result, confirming that PV transformation improved the predictive ability of the DR prediction model. Figure 7 also shows that the samples are distributed closer to the diagonal line than those in Figures 3 and 4, indicating that the DR can be predicted accurately. In particular, we succeeded in reducing the number of samples for which the DR was estimated to be small and the actual DR was large. In the multivariate analysis, we confirmed that the predictive ability of the machine learning model can be improved by appropriately transforming x such that it is related to y.

The feature importance was calculated for the LGB model in Case Study 3, which had the highest predictive ability, as shown in Table 2. The feature importance of E* was the highest, confirming that the proposed variables and variable transformations improved the predictive ability of the model. We confirmed that the actual management of E* successfully reduced the DR and that machine learning can be used to construct an appropriate DR prediction model and identify PVs related to the DR.

**TABLE 2:** Feature importance in the LightGBM model.

A	0.07
B	0.17
C	0.07
D	0.03
E	0.19
E*	**0.23**
F	0.15
F*	0.09

The data analysis in this study confirmed that machine‐learning‐based models can contribute to process improvement using routinely measured process data, including the data of PVs that were not considered important in the process, and that the predictive ability of machine‐learning models can be improved by utilising domain knowledge of the process. Machine learning cannot extract significant information without the necessary data. However, it can construct appropriate models even with unnecessary data. Even the PV data that may not be relevant to the process can be added and considered for machine learning.

In this study, machine learning was used to predict the DR in a mass‐production process for precision electrical components, and PVs related to the DR were identified. In a dataset with PVs that were not correlated with the DR in univariate analysis, we confirmed that a multivariate analysis improved the correlation coefficient between the actual and predicted DRs in the test data. The predictive ability of the model was improved by adding PVs that were not originally considered important, and a further improvement in the predictive ability was achieved by appropriately transforming and analysing the PVs according to on‐site conditions. The LGB model enabled the construction of the best DR prediction model. By checking the feature importance in the model, we succeeded in diagnosing PVs related to the DR. The proposed method can be applied to routinely measured data and machine‐learning models can be constructed based on the data obtained using this method. In addition, it was confirmed that it is important to use domain knowledge to improve the prediction ability of machine learning models and interpret the constructed models. This research can contribute to the prediction of DRs for various processes and the diagnosis of related PVs.

## CONFLICT OF INTEREST STATEMENT

The author declares no conflict of interest.

## Data Availability

Data are available upon request.
